# Antibiotic Resistance in the Philippines: Environmental Reservoirs, Spillovers, and One-Health Research Gaps

**DOI:** 10.3389/fmicb.2025.1711400

**Published:** 2025-12-12

**Authors:** Charmaine Ng, Josephine Abrazaldo, Patrick de Vera, Shin Giek Goh, Boonfei Tan

**Affiliations:** 1 Institutional Research Office, Manila Central University, Caloocan, Philippines; 2 College of Medical Technology, Manila Central University, Caloocan, Philippines; 3 Environmental Health Institute, National Environment Agency, Singapore, Singapore

**Keywords:** antimicrobial resistance, One Health, hospitals, wastewater treatment plants, environmental waters, food, agriculture, sewage

## Abstract

Antimicrobial resistance (AMR) remains a major health threat in the Philippines, where high antimicrobial use, intensive aquaculture, and recurrent typhoon-driven flooding and monsoon seasons shape distinctive transmission pathways. This narrative review synthesizes published Philippine data across clinical, agriculture, and environmental sectors to map evidence and gaps relevant to policy. Clinically, vancomycin resistant *Enterococcus faecium* is 23%, *Klebsiella pneumonia* shows ∼15% carbapenem resistance, and *Escherichia coli* resistance to third generation cephalosporins (3GC) and fluoroquinolone are ∼43 and ∼46%, respectively. In food animals, ceftriaxone resistance in non-typhoidal *Salmonella* (NTS) increased from ∼8% (2010) to ∼43% (2020s), with ciprofloxacin resistance between 14 and 23%. Environmental studies report extended spectrum beta-lactamase (ESBL)-producing *E. coli* in Manila estuaries and multiple antibiotic resistance (MAR) indices of up to 0.15 in tributaries. Hospital sewage and nearby rivers have yielded carbapenemase-producing Enterobacterales (CPE) bearing *bla*_NDM_/*bla*_KPC_ in clinically relevant lineages (e.g., *E. coli* CC10, *K. pneumoniae* ST147). Cross sector comparisons remain constrained by method heterogeneity and data gaps. To operationalize One-Health monitoring, we propose (i) a two window surveillance design: late dry baseline and 24–72 h post-storm flood pulses; and (ii) a two tier analytics model: Tier 1 HT-qPCR ARG/MGE panels at regional hubs for rapid screening, and Tier 2 metagenomics/isolate whole genome sequencing (WGS) at national hubs for source attribution and plasmid tracking. We translate these findings into a modular AMR risk assessment toolkit to prioritize surveillance and targeted interventions.

## 1 Introduction

Rivers, sediments, and wastewater networks function as persistent environmental reservoirs of AMR, concentrating and exchanging antibiotic resistant bacteria (ARB) and antibiotic resistance genes (ARGs) across human, animal, and environmental interfaces (Aslam et al., 2021; Fouz et al., 2020; Grenni, 2022; Larsson and Flach, 2022). In Southeast Asia (SEA), AMR selection and dissemination are driven by untreated urban sewage, effluents from wastewater treatment plants (WWTPs), and runoffs from agricultural and aquaculture (Anh et al., 2021; Siri et al., 2023). Philippine rivers and estuaries sit at the nexus of an archipelagic, typhoon-exposed water network which amplifies the threat of AMR dissemination in water sources. A recent global fate model estimates that ∼29% of the forty most used human antibiotics (e.g., amoxicillin, sulfamethoxazole, ciprofloxacin) ultimately reach rivers, with extensive stretches of SEA, including the Philippines, exceeding ecosystem-protection and resistance-selection thresholds under low-flow conditions (Ehalt Macedo et al., 2025). Typhoons, monsoon rains and flood pulses are among the country’s most frequent hazards, periodically connecting sewage, surface waters, and sediments, resuspending pathogens and AMR vectors along human-water-food pathways (PAGASA, 2024; Tan et al., 2025). Climate change (stronger cyclones, heavier rainfall extremes, sea-level rise/backflow, and warming) intensifies these hydrologic linkages and heightens AMR transmission risk (IPCC, 2022; Tan et al., 2025).

Within this setting, the National Capital Region of the Philippines, Metro Manila, is vulnerable as it is a delta megacity (population density ∼21,000 persons/km^2^) straddling the Pasig river and Manila Bay mixing chamber. It faces chronic wastewater and infrastructure management challenges with only a fraction of sewage captured and treated (World-Bank, 2020, 2024). The metropolis has a high concentration of tertiary/specialty hospitals and large informal makeshift settlement communities (i.e., squatter areas, shanty towns, slums) with national estimates of ∼500,000 informal settler families residing along waterways (International-Trade-Administration, 2024; UN-Habitat, 2015). Many districts rely on combined drainage which results in mixing and co-discharge of stormwater and untreated sewage to environmental surface waters. This provides a direct pathway for pathogens, ARB, antibiotics, and ARGs to enter natural waterways, with clinical strains of ARB documented in surface waters (Suzuki et al., 2020; World-Bank, 2020). Limited sewage coverage, aging infrastructure, and frequent typhoon/monsoon stormwater flooding drive periodic sewer overflows, flushing pathogens, antibiotics, pharmaceutical residues, heavy metals, ARB, and ARGs from hospitals, households, farms, and industries, into rivers and estuaries creating public-health risks across the urban-coastal continuum.

Complementing these urban pathways, the agricultural economy adds parallel selection and dissemination routes for AMR. In the Philippines, the agriculture sector contributes ∼9% of the national gross domestic product (GDP), with livestock, poultry, and crop together accounting for > 70% of agricultural output (Philippines-Statistics-Authority, 2019). Over-the-counter access to veterinary antimicrobials, prophylactic use, partial courses and limited veterinary oversight have been documented, and are among the practices associated with the burden of antibiotic resistant *E. coli*, *Salmonella*, and Enterobacterales in swine and poultry value chains (Barroga et al., 2020; Gundran et al., 2020; Gundran et al., 2019; Lim et al., 2017; Ng and Rivera, 2014). In aquaculture, antibiotic use and pond-sediment/effluent management creates ARG reservoirs in shrimp and tilapia systems, with routine water exchange and post-harvest cleaning discharging residues, ARB, and ARGs into canals, rivers, and near shore fisheries (FAO, 2021; Suyamud et al., 2024; Tendencia and de la Peña, 2001). Co-selection by metals and biocides in pond environments further maintains resistance even when direct antibiotic pressure is low (Pal et al., 2015; Wales and Davies, 2015). Monsoon and flood pulses rapidly mixes these aquaculture effluents with municipal waters, accelerating dissemination (Tan et al., 2025). These agri-food flows link to wet markets and slaughter/animal processing nodes, enabling environment-to-food spillover and feedback of farm/animal adapted AMR strains into communities (Grace, 2015; Nhung et al., 2016).

This narrative review addresses synthesizes peer-reviewed and gray literature on AMR in the Philippines. We searched Pubmed, Web of Science, and Google Scholar and screened gray sources from the Department of Health (DOH), Antimicrobial Resistance Surveillance Reference Laboratory (ARSRL), Antimicrobial Resistance Surveillance Program (ARSP), WHO/FAO/OIE, UN-Habitat, World Bank, PAGASA, IPCC (Food and Drug Administration, 2025; Department of Health Pharmaceutical Division, 2019), Department of Agriculture (DA), Bureau of Animal Industry (BAI), and Bureau of Fisheries and Aquatic Resources (BFAR, 2024). Search strings combined terms for antimicrobial resistance/antibiotics; Philippines/Metro Manila/SEA; hospitals/clinical; agriculture/aquaculture/food; wastewater/rivers/estuaries/coastal; flood/typhoon/monsoon/climate change; surveillance/WHONET, genomics/metagenomics/HT-qPCR; and risk assessment. We included English language articles and surveillance reports from the past 12 years but retained a few sentinel papers.

We compare antibiotic use and governance in hospitals and farms, and chart ARB trends across clinical, food, and environmental reservoirs to identify priority pathogens and data gaps. We trace pathways by which hospital effluent and community wastewater reach rivers, estuaries, and coastal waters, and discuss drivers of AMR persistence and dissemination (selection, co-selection, mobilization, transport), taking into consideration how climate hazards and mixed land use tie these systems together, particularly through flood pulses and urban-coastal linkages. Building on this evidence, we propose strategies for environmental monitoring of AMR to close data gaps, AMR risk assessment frameworks as a policymaking toolkit, and recommended priority actions aligned to the Philippines National Action Plan (PNAP) to support surveillance, stewardship and infrastructure action.

## 2 Antibiotic usages in hospitals and the farming industries

Since 2017, the WHO introduced the AWaRe (Access Watch Reserve) framework to guide stewardship by classifying antibiotics into Access (first/second line treatments, lower resistance potential), Watch (higher resistance potential) and Reserve (last-resort treatment) categories (WHO, 2025a). The overall prevalence of antimicrobial use in private and public hospitals in the Philippines was 56–58% from 2019 to 2021 (De Los Reyes et al., 2023). Global Point Prevalence Surveys in local hospitals (2017–2019) showed prescribing dominated by Watch antibiotics (72–5%, mainly piperacillin-tazobactam and cefuroxime), followed by Access (∼23–26%), and Reserve (∼1–2%) drugs (de Guzman Betito et al., 2021). Contributing to this pattern include poor treatment adherence, prescription-sharing among patients, weak supply chain integrity, inappropriate treatment regimes, and lack of training and treatment monitoring (DOH-RITM, 2023). Notably, Global Point Prevalence Surveys reported ceftriaxone (14–16%) and piperacillin-tazobactam (13–15%) as the most used antibiotics for pneumonia/COVID from 2019 to 2021 (De Los Reyes et al., 2023). Table 1 summarizes the most used antibiotics (piperacillin-tazobactam, ceftriaxone, meropenem, vancomycin, levofloxacin) in hospitals, the AWaRe classifications, and route of administration drawing from available national surveillance data and published reports (2017–2021) (Abeleda et al., 2024; de Guzman Betito et al., 2021; De Los Reyes et al., 2023; De Los Reyes et al., 2025, 2019; DOH-RITM, 2023).

**TABLE 1:** Most commonly used antibiotics in hospitals, farm/food animal and aquaculture sectors in the Philippines.

Hospitals^1^				
Antibiotics	Drug class	AWaRe classification^4^	Route	Oversight^5,6,7^
Piperacillin-tazobactam	Extended-spectrum ureidopenicillin + beta-lactamse inhibitor	Watch	Intravenous	WHO EML; FDA PH: Regulated; PNF: Stewardship controls
Ceftriaxone	3GC	Watch	Intravenous, Intramuscular	WHO EML; FDA PH: Regulated; PNF: Stewardship controls
Meropenem	Carbapenem (beta-lactam)	Watch	Intravenous	WHO EML; FDA PH: Regulated; PNF: Restricted antimicrobial (IV)
Vancomycin	Glycopeptide	Watch	Intravenous; oral	WHO EML; FDA PH: Regulated; PNF: Restricted antimicrobial (IV)
Levofloxacin	Fluoroquinolone	Watch	Intravenous; oral	WHO EML; FDA PH: Regulated; PNF: Stewardship controls
**Farm/food animals^2^**				
**Antibiotics**	**Drug class**	**AWaRe classification^4^**	**Route**	**Oversight^6,8^**
Tylosin	Macrolide	Not classified (veterinary use only)	Poultry: via medicated water Swine: Intramuscular	FDA PH: Regulated (veterinary prescription only); DA-BAI: farm AMU monitoring and compliance
Oxytetracyclin	Tetracycline	Not classified (veterinary use only)		FDA PH: Regulated (veterinary prescription only); DA-BAI: farm AMU monitoring and compliance
Amoxicillin	Penicillin (beta-lactam)	Access		FDA PH: Regulated (veterinary prescription only); DA-BAI: veterinary AMU monitoring and compliance
Enrofloxacin	Fluoroquinolone	Not classified (Veterinary use only)		FDA PH: Regulated (veterinary prescription only); DA-BAI: AMU monitoring
Colistin	Polymyxin	Reserve		WHO: Highest Priority Critically Important Antimicrobial FDA PH: Regulated (veterinary prescription only); DA-BAI: AMU monitoring and advisories against non-therapeutic use
**Aquaculture^3^**				
**Antibiotics**	**Drug class**	**AWaRe classification^4^**	**Route**	**Oversight^6,8,9^**
Oxytetracyclin	Tetracycline	Access	Medicated feed, immersion (fish and shrimp)	FDA PH: Regulated (veterinary prescription only; BFAR, DA-BAI: AMU monitoring in veterinary channels
Erythromycin	Macrolide	Access	Oral feed, injection (in tilapia farms for streptococci infections)	FDA PH: Regulated (veterinary prescription only; BFAR, DA-BAI: aquaculture compliance/AMU monitoring
Sulfonamides	Sulfadrugs	Access	Oral feed, immersion	FDA PH: Regulated (veterinary prescription only; BFAR, DA-BAI: aquaculture compliance/AMU monitoring
Enrofloxacin	Fluoroquinolone	Watch	Oral feed, injection	FDA PH: Regulated (veterinary prescription only; BFAR, DA-BAI: aquaculture compliance/AMU monitoring
Florfenicol	Amphenicol	Watch	Oral feed, immersion	FDA PH: Regulated (veterinary prescription only; BFAR, DA-BAI: AMU monitoring in veterinary channels

WHO EML, WHO Model list of essential medicines; FDA PH, FDA Philippines; PNF, Philippine National Formulary; DA, Department of Agriculture; BAI, Bureau of Animal Industry; AMU-Antimicrobial Usage; BFAR, Bureau of Fisheries and Aquatic Resources. References:

^1^(Abeleda et al., 2024; de Guzman Betito et al., 2021; De Los Reyes et al., 2023; De Los Reyes et al., 2025, 2019; DOH-RITM, 2023);

^2^(Barroga et al., 2020);

^3^(Bornales and Tahiluddin, 2025);

^4^(WHO, 2025a);

^5^(WHO, 2025b);

^6^(FDA PH: Food and Drug Administration, 2025);

^7^(PNF: Department of Health Pharmaceutical Division, 2019);

^8^(DA/BAI, 2025);

^9^(BFAR, 2024).

The Philippines, like many low and middle income countries (LMICs) face challenges in regulating antimicrobial use in the farming industry due to weak enforcement or prescription policies of veterinary medicinal products (VMP) (Barroga et al., 2020). With crops (49%), and livestock/poultry production (25%) comprising the bulk of the national agriculture production, rising demand has driven intensive swine and poultry farming (Philippines-Statistics-Authority, 2019). Food safety and AMR-related health risk have been detected in food sources such as vegetables, swine and poultry meat in the Philippines (Calayag et al., 2021; dela Peña et al., 2022; Gundran et al., 2020; Gundran et al., 2019; Lim et al., 2017; Ng and Rivera, 2014; Vital et al., 2018). A biosecurity study in 2018 found that 44–81% of farms used one to two antimicrobials (Barroga et al., 2020). In the same study, the most prescribed antibiotic classes reported in swine farms (*n* = 54) were fluroquinolones (enrofloxacin 36%), tetracyclines (oxytetracycline 30%) and macrolides (tylosin 25%) (Barroga et al., 2020). Poultry farms (*n* = 39) commonly used fluroquinolone (norfloxacin 25%), (fosfomycin 10%), and (oxytetracycline 10%) (Barroga et al., 2020). By production type, commercial farms (*n* = 60), most frequently applied enrofloxacin (40%), amoxicillin (30%), tiamulin (17%), and colistin (17%), while backyard farms (*n* = 33) relied mainly on oxytetracycline (39%), enrofloxacin (24%), and doxycycline (15%) (Barroga et al., 2020). Respiratory diseases were the most common reason for antibiotic use with enrofloxacin applied broadly for enteric, respiratory, and non-specific conditions (Barroga et al., 2020). Notably, several of these antibiotics overlap with those critical in human medicine, raising public health concerns. Table 1 provides an overview of the top 5 antibiotics used in farm animals in the Philippines. Antibiotic use in Philippine aquaculture is commodity-specific and central to disease management, with implications for both productivity and environmental health. Shrimp farms commonly use oxytetracycline, enrofloxacin, sulfonamides to control bacterial infections like vibriosis, typically via medicated feeds or pond applications (Bornales and Tahiluddin, 2025). Tilapia production relies on a combination of oxytetracycline, florfenicol, and sulfa-trimethoprim to manage streptococcosis and other infections, while milkfish farms often employ oxytetracycline and quinolones (e.g., norfloxacin, ciprofloxacin) in pond and caged systems (Tahiluddin et al., 2025; Table 1). Beyond antibiotics, aquaculture uses antiparasitic agents, pesticides, antifungals, and disinfectants to reduce mortality and boost production (Tahiluddin et al., 2025). Residues of tetracycline and quinolone have been detected in sediments, rivers, and coastal waters near aquaculture sites, pointing to environmental accumulation and AMR selection pressures (Tahiluddin et al., 2025). Large-scale commercial shrimp farms tend to use broad-spectrum antibiotics more frequently, while small tilapia and milkfish farms depend heavily on prophylactic medicated feed (Regidor et al., 2020). Collectively, these findings indicate that antibiotics are integral to aquaculture disease management, however strengthened monitoring systems, stronger veterinary oversight, and adoption of alternative measures (e.g., vaccination, probiotics) should be considered to reduce risks of resistance dissemination across aquatic and human health interfaces.

## 3 Mapping AMR trends in pathogens: clinical, food, and environmental reservoirs

ESKAPE pathogens are a group of multidrug resistant (MDR) bacteria (*E. faecium*, *Staphylococcus aureus*, *K. pneumoniae*, *Acinetobacter baumannii*, *Pseudomonas aeruginosa*, and *Enterobacter* spp.), that drive nosocomial infections and are a significant public health threat due to their ability to evade treatment by antibiotics (Miller and Arias, 2024). In the Philippines, the ARSRL under the ARSP tracks clinical resistance trends in priority clinical pathogens through systematic data collection, laboratory validation, and reporting (ARSRL, 2025). While this framework is well established in hospital and clinical settings, surveillance in other One Health sectors such as animal production, food safety, and environmental reservoirs remain fragmented, creating critical blind spots in identifying drivers of resistance beyond human health. To address this gap we anchored our analysis to the 2024 ARSRL baseline dataset and compared resistance rates from published studies across One Health sectors to gain a broader perspective of AMR trends in the Philippines (Table 2). Importantly, this list is not exhaustive and may not capture all ESKAPE organisms or their resistance profiles reflecting limitations in current surveillance and the need for expanded cross sector monitoring.

**TABLE 2:** A comparison of antibiotic resistance profiles of opportunistic pathogens in clinical (human source), food (animal/crop sources) and environmental (soil, water) settings in the Philippines.

Percentage resistance	Clinical (Human)	Food (animals, crop)				Environmental (soil, water)			
Period	2024	NR	2017	<2018	2017-2019	2016-2018	<2018	2017-2019	2024
*E. coli*	(n = 14,894)	(n = 162)	(n = 78)	(n = 26)	(n = 142)	(n = 11)	(n = 212)	(n = 450)	(n = 48)
Source	Hospital infections (1)	Pigs anal & boot swab (2)	Chicken anal swab (3)	Vegetables (4)	Animal & Human faecal source (5)	Hospital & rivers (6)	Irrigation waters & soil (4)	Rivers & tributaries (5)	Estuaries (7)
AST methodology, guidelines	Vitek 2 compact, E-test, disc diffusion, CLSI	Vitek 2 Compact, CLSI	Vitek 2 Compact, CLSI	Disc diffusion, CLSI	Vitek 2 Compact, CLSI	Enriched on CHROMagar mSuperCARBA, agar dilution, CLSI	Disc diffusion, CLSI	Vitek 2 Compact, CLSI	Vitek 2 Compact, CLSI
Carbapenem	7.0-9.0	0	1.5 −2.9	ND	ND	54.5-63.6	ND	0.4	<5
Cefotaxime	42.1	ND	ND	3.8	ND	ND	10.3	ND	ND
Ceftriaxone	38.4	97.9	95.7	ND	ND	ND	ND	1.3	<5
Cefepime	17.8	93.7	82.6	ND	ND	ND	ND	0.9	ND
Cefoxitin	18.7	25	46.4	ND	ND	ND	ND	ND	<5
Ceftazidime	25.3	100	97.1	ND	ND	ND	ND	ND	54.2
Co-trimoxazole	52.1	89.6	72.5	ND	31.7	ND	ND	28.2	45.8
Ciprofloxacin	45.7	43.8	91.3	7.6	10.6	ND	7.8	7.7	16.7
Amoxicillin-clavulanic acid	22.8	12.5	43.5	ND	ND	ND	ND	ND	<10
Piperacillin-tazobactam	17.5	2.1	20.3	ND	ND	ND	ND	1.1	<5
Colistin	2.3	4.2	8.7	ND	ND	ND	ND	ND	ND
Chloramphenicol	ND	ND	ND	7.6	ND	ND	17.2	ND	ND
Tetracycline	50.5	ND	ND	42.3	ND	ND	38.6	ND	ND
**Percentage resistance**	**Clinical (Human)**	**Food (animals, crop)**				**Environmental (soil, water)**			
**Period** ***K. pneumoniae***	**2024** **(n = 18,378)**					**2016-2018** **(n = 14)**	**2021** **(n = 12)**	**2023** **(n = NR)**	
**Source** **(Reference)**	**Hospital infections (1)**					**Hospital & rivers (6)**	**Coastal waters (8)**	**Aquaculture River & Sediment (9)**	
**Methodology,** **guidelines**	**Vitek 2 compact, E-test, disc diffusion, CLSI**					**Enriched on CHROMagar mSuperCARBA, agar dilution, CLSI**	**Vitek 2 Compact, CLSI & EUCAST**	**Disc diffusion, CLS**	
3GC	39.7-45.8					ND	8.0	*Klebsiella* spp. accounted for 10-60% of isolated bacteria, resistance profile unclear	
Carbapenems	15.7-16.4					57.1	ND		
Fluoroquinolones	26.4-43.2					ND	ND		
Piperacillin-tazobactam	37.4					ND	ND		
**Period** ***A. baumannii***	**2024** **(n = 6,683)**					**2023** **(n = NR)**			
**Source (Reference)**	**Hospital infections (1)**					**Aquaculture River & Sediment (9)**			
**Methodology, guidelines**	**Vitek 2 compact, E-test, disc diffusion, CLSI**					**Disc diffusion, CLSI**			
3GC	47.8-50.8					*Acinetobacter* spp. accounted for 10% of isolated bacteria, resistance profile unclear			
Carbapenems	49.2-49.6								
Ampicillin-sulbactam	42.0								
Piperacillin-tazobactam	51.0								
Fluoroquinolones	46.6-47.1								
Colistin	0.4								
**Percentage resistance**	**Clinical (Human)**	**Food (animals, crop)**				**Environmental (soil, water)**			
**Period**	**2024**					**2024**			
***K. faecalis***	**(n = 3,714)**					**(n = 18)**			
**Source (Reference)**	**Hospital infections (1)**					**Irrigation waters** **from urban farms** **(10)**			
**Methodology, guidelines**	**Vitek 2 compact, E-test, disc diffusion, CLSI**					**Broth microdilution, CLSI**			
Gentamicin	17.2					*Enterococci* spp. with low resistance to ciprofloxacin (6%)			
Streptomycin	16.6								
Vancomycin	2.8								
Linezolid	2.7								
**Period**	**2024**								
***E. faecium***	**(n = 1,931)**								
**Source (Reference)**	**Hospital infections (1)**								
**Methodology, guidelines**	**Vitek 2 compact, E-test, disc diffusion, CLSI**								
Penicillin	92.6								
Ampicillin	89.8								
Vancomycin	22.6								
Nitrofurantoin	50.4								
**Percentage resistance**	**Clinical (Human)**	**Food (animals, crop)**				**Environmental (soil, water)**			
**Period**	**2024**	**2013-2014**	**2018-2022**	**2022**					
	**(n = 351)**	**(n = 178)**	**(n = 105)**	**(n = 95)**					
*Salmonella spp.*	**Non-typhoidal *Salmonella spp.* (NTS)**	***S. enterica***	***S. enterica***	***S. enterica***					
**Source (Reference)**	**Hospital infections (1)**	**Pig meat (11)**	**Chicken, Pig meat (12)**	**Chicken meat (13)**					
**Methodology, guidelines**	**Vitek 2 compact, E-test, disc diffusion, CLSI**	**Vitek 2 compact, CLSI**	**Vitek 2 compact, CLSI**	**Vitek 2 compact, CLSI**					
Ceftriaxone	19.2	7.9	24.0	43.2					
Ciprofloxacin	9.8	15.7	23.0	13.7					
Meropenem	2.4	0	0	0					
Co-trimoxazole	14.1	70.8	27.0	11.6					

AMR profiles of specific opportunistic pathogens were compiled from published studies and reports available over the last 10 years. ND, no data available; NR, not reported; AST, Antibiotic susceptibility testing; CLSI, Clinical and Laboratory Standards Institute; EUCAST, European Committee on Antimicrobial Susceptibility Testing. References:

^1^(DOH-RITM, 2025),

^2^(Gundran et al., 2020),

^3^(Gundran et al., 2019),

^4^(Vital et al., 2018),

^5^(dela Peña et al., 2022),

^6^(Suzuki et al., 2020),

^7^(Palmares et al., 2024),

^8^(Cardos et al., 2023),

^9^(Mendoza and Reyes, 2024),

^10^(Oliveros and Vital, 2022),

^11^(Calayag et al., 2021),

^12^(Mora et al., 2023),

^13^(Nagpala et al., 2025).

### 3.1 E. coli

**Clinical:** 3GC < 43%, co-trimoxazole 52%, ciprofloxacin 46%, carbapenems < 9% (DOH-RITM, 2025). Regional comparator (Indonesia): Higher: 3GC/trimethoprim-sulfamethoxazole/fluoroquinolones > 60%; carbapenems ∼18% (Gach et al., 2024).

**Food animals:** Broiler carbapenem resistance 1.5–2.9%; farm reared pigs/chickens high: 3GC 25–100%, co-trimoxazole 73–90%, ciprofloxacin 44–91%; colistin 4–9% (clinical ∼2%) (Gundran et al., 2019; Suzuki et al., 2020). Regional comparator (Thailand): Higher: colistin 41–73% in sick pigs carrying *mcr-1* genes (Trongjit et al., 2022).

**Environment:** Manila estuaries: ciprofloxacin 17%, 3GC < 5%, carbapenems < 5%; hospital sewage/river 55–64% (likely inflated by CHROMagar supplemented with beta-lactam antibiotics) (dela Peña et al., 2022; Palmares et al., 2024; Suzuki et al., 2020).

**Implication and recommendations:** Food animals are major *E. coli* AMR reservoirs; prioritize (i) harmonized antibiotic susceptibility testing (AST) across sectors, (ii) colistin/*mcr* surveillance in farms/slaughterhouses, and (iii) detect source attribution using whole genome sequencing (WGS) to link animal, clinical, and wastewater isolates.

### 3.2 K. pneumoniae

**Clinical:** 3GC ∼40–45%, carbapenems 15%, fluoroquinolones 26–43% (DOH-RITM, 2025). Regional comparator (Indonesia): Higher: 3GC 74%, carbapenems 22%, fluoroquinolones 53% (Gach et al., 2024).

**Food animals:** ND/NR.

**Environment:** Limited, heterogeneous methods used and not directly comparable to clinical datasets (Cardos et al., 2023; Mendoza and Reyes, 2024; Suzuki et al., 2020).

**Implication and recommendations:** Close the cross-sector gap by (i) conducting animal (farms/slaughterhouses) and anthropogenically impacted environmental sentinel sampling sites, (ii) harmonizing AST across sectors, (iii) WGS to detect spillover routes and plasmid typing for ARG dissemination.

### 3.3 Acinetobacter baumannii

**Clinical:** Approximately 50% resistance across 3GC, carbapenems, beta-lactam/beta-lactamase inhibitors, and fluoroquinolones, colistin 0.2% (DOH-RITM, 2025). Regional comparator (Indonesia): Higher: carbapenems 71%, colistin 48% (Gach et al., 2024)

**Food animals:** ND/NR.

**Environment:** Sparse and under-represented (Mendoza and Reyes, 2024).

**Implication and recommendations:** Carbapenem resistant *A. baumannii* remains a critical threat clinically; urgent need for non-clinical sectors to conduct baseline work. Implement baselines in the food and environmental sector implementing aforementioned methods.

### 3.4 *Enterococci* spp.

**Clinical:** Vancomycin resistant *E. faecalis* 3%, *E. faecium* 23% (DOH-RITM, 2025). Regional comparator (South East Asia): Vancomycin resistance *Enterococci* spp. (VRE) ∼12 % (Huang et al., 2025).

**Food animals:** ND/NR.

**Environment:** Only one irrigation water study: ciprofloxacin 6%, no VRE detected (Oliveros and Vital, 2022).

**Implication and recommendations:** Clinically high *E. faecium* VRE detected, however lack of monitoring data across food and environmental sectors. Global evidence of reservoirs in poultry (15–16%) and livestock workers (11%) indicates zoonotic transmission risk (Caddey et al., 2025), Prioritize standardized food and environmental surveillance work in the Philippines, particularly species resolved surveillance in poultry/swine value chains, and genotyping to enable food-environment-human source tracing.

### 3.5 S. enterica

**Clinical:** 3GC (ceftriaxone) ∼19%, ciprofloxacin ∼10% (DOH-RITM, 2025).

**Food animals:** In pigs/chicken: ceftriaxone resistance rising (∼8% in 2010s to ∼43% in 2020s); ciprofloxacin 14–23%; co-trimoxazole resistance reduction (∼71% in 2010s to ∼12-27% in 2020s) (Calayag et al., 2021; Mora et al., 2023; Nagpala et al., 2025).

**Environment:** ND/NR.

**Implication and recommendations:** Salmonella is a leading foodborne pathogen transmitted primarily via contaminated food and water (Lamichhane et al., 2024). Resistance to 3GC such as ceftriaxone, often mediated by plasmids encoding ESBL genes (e.g., *bla*_CTX,_ *bla*_TEM_), is increasingly detected in NTS from food animals (Alvarez et al., 2023; Matsumoto et al., 2014; Pouget et al., 2013). Food animals are key

*S. enterica* MDR reservoirs with spillover risk; necessitatinga the need to strengthen the farm-to-clinic linkage. Integrate AMR surveillance studies (i) on farms/slaughterhouses, animal meats sold at local markets, (ii) aquatic environments that receive runoffs from animal source facilities, (iii) track ESBL/fluoroquinolone plasmids with WGS to map farm-to-clinic/environment flow, and (iv) target interventions at high risk nodes.

## 4 Hospital to environment: AMR spillovers and environmental reservoirs in the Philippines

Leakage of ARB and ARG from hospitals to waterways has been documented in the Philippines. Between 2016 and 2019, CPE (*E. coli* CC10 and *K. pneumoniae* ST147) carrying *bla*_NDM,_ and *bla*_KPC_ were isolated from hospital sewage and adjacent rivers in the Philippines. Conjugation assays confirmed plasmid mediated gene transfer in 24 isolates (Suzuki et al., 2020). In five of the seven hospitals with on-site wastewater treatment trains, CPE appeared only in pre-treated effluents (Suzuki et al., 2020). Cross sectional studies and intermittent sampling limit causal inference of whether flood-pulse dynamics or sewer leakages contribute to hospital to river spillover.

Environmental studies further highlight the persistence of AMR. From 2017 to 2019, *E. coli* from Laguna Lake catchment showed resistance to 14 of 16 antibiotics tested, with highest resistance to ampicillin (44%), and a maximum multiple antibiotic resistance (MAR) index of 0.15 at Pila River (dela Peña et al., 2022). Tributaries including Biñan, San Cristobal, and Sta. Rosa which receive WWTP effluents were major contributors (dela Peña et al., 2022). More recently, a 2024 study of Manila estuaries, showed an average of 4.3 × 10^4^ CFU of *E. coli*/100 mL, with 15% of isolates classified as MDR (Palmares et al., 2024). Among these, 6% were ESBL producers, and 19% exhibited a MAR index of ≥ 0.2, indicating a high-risk contamination hotspots (Palmares et al., 2024). By implementing storm-event, high frequency samplings (using autosamplers with flow-weighted composites), paired with rainfall/river data, and source-tracking genomics, AMR flood-driven pulse spillovers can be separated from attribution to continuous sewer leakages.

## 5 Drivers and mechanisms of AMR persistence in the environment

AMR persistence is shaped by both chemical and microbiological factors. The Philippine river–coast continua carry parts-per-billion (ppb) of pharmaceuticals at levels sufficient for resistance selection below clinical minimal inhibitory concentrations (MICs), and near proposed predicted no-effect concentrations (PNECs). For example, sulfamethazine has been detected at up to 764.91 ppb near Macajalar Bay, while ciprofloxacin (9.84–92.24 ppb), sulfamethoxazole (129.55–470.87 ppb), and azithromycin (38.84–223.89 ppb) have been detected in untreated hospital wastewater during dry season when dilution is low (Mariano et al., 2023). AMR can persist in the absence of antibiotic exposure, sustained by environmental contaminants such biocides/disinfectants (e.g., triclosan, benzalkonium chloride), and heavy metals. For example, microplastics, and heavy metals (i.e., iron, zinc, lead, cadmium) have been detected in sediments of Pasig river particularly in areas of high urbanization during the wet season (Ilao et al., 2025). Heavy wet-season rains wash pollutants from roads, landfills, industrial sites, and household waste into rivers, where they accumulate in riverbed sediments (Ilao et al., 2025). These pollutants exert selective pressure, with ARGs often co-localized with biocide and metal resistance genes (BMRGs) on the same plasmid, promoting co-selection and horizontal gene transfer (HGT) (La Rosa et al., 2025; Pal et al., 2015).

In parallel, biofilms in WWTPs, riverbeds, and on microplastics act as gene-exchange hubs where conjugation, transformation, and transduction occur more frequently than in planktonic communities. These biofilm structures not only stabilize imported clinical resistance traits but facilitates HGT under sub-MIC conditions (Gross et al., 2025; Madsen et al., 2012; Michaelis and Grohmann, 2023). In WWTPs worldwide, biofilms known to stabilize carbapenemase genes (*bla*_NDM_, *bla*_KPC_) and are hotspots for HGT (Ng et al., 2017; Oliveira et al., 2021; Tiwari et al., 2022; Zhang et al., 2009). In the Philippines, environmental Enterobacteriaceae carrying plasmids encoding for carbapenem resistance genes have been detected in hospital sewage and rivers, highlighting direct clinic to environment leakage. Furthermore, conjugative transfer of these plasmids have been demonstrated (Suzuki et al., 2020). Riverbed biofilms have been shown to harbor ESBL-producing *E. coli* and carbapenemase genes in freshwater sediments elsewhere (Reichert et al., 2021; Skof et al., 2024). Microplastic-associated biofilms in coastal waters also promote ARG persistence; for example, microplastics have been reported to increase conjugation frequencies of up to 20-fold and to enrich ARGs such as *mcr-2* and *bla*_CTX–M_ (Tang and Li, 2024; Zhou et al., 2024). Together, these findings illustrate how pollution inputs from hospital effluents to plastic debris could interact to reinforce AMR persistence across Philippine aquatic systems.

## 6 Climate hazards, mixed land-use, and AMR dissemination

Climate hazards further amplify AMR dissemination in ways that are distinct to an archipelago with infrastructure gaps. The Philippine Atmospheric, Geophysical and Astronomical Servies Administration (PAGASA) projects mid-century warming of 0.9–1.9°C under Representative Concentration Pathway (RCP) 4.5 (moderate warming) and 1.2–2.3°C under RCP8.5 (more severe warming). These changes will be accompanied by wetter wet seasons and drier dry seasons, conditions that influence microbial growth, dilution dynamics, and transport pathways of ARB and ARGs (PAGASA, 2018). The Philippines experiences nearly 20 typhoons annually, making it one of the most hazard prone countries in the world with frequent flooding. During typhoons and floods, stormflow and combined-sewer overflows mobilize sewage, raising ARG levels and sewage markers downstream for hours (Garner et al., 2017; La Rosa et al., 2025; Sojobi and Zayed, 2022). Metro Manila is particularly vulnerable given its mixed drainage system and frequent flood pulses. Flood-associated outbreaks illustrate this risk where gastroenteritis surged 7.5 fold in Kanaga, Leyte following Typhoon Haiyan in 2013, linked to water contaminated by *Aeromonas hydrophila* (Ventura et al., 2015); and during the 2009 Metro Manila floods, a leptospirosis outbreak caused 471 hospitalizations and 51 deaths, indicating extensive sewage contact and system breaches during inundation (Amilasan et al., 2012). Urban sewage, industrial effluents, and agricultural runoff converge within shared catchments creating multiple pathways of contamination. Regional reviews across East and Southeast Asia identify aquaculture and livestock wastewater, together with untreated sewage and WWTP effluents as major AMR contributors (Larsson and Flach, 2022; Shimizu et al., 2013; Suzuki and Hoa, 2012). This pattern is evident in Metro Manila and Laguna Lake tributaries (2017–2019), where fecal contamination and resistant *E. coli* occur alongside WWTP discharges and human sewage signals; where chicken fecal isolates showed a MAR index of 0.17 and water at Pila River water at 0.15 (dela Peña et al., 2022). In coastal zones (Davao Gulf; Macajalar Bay) ppb-level pharmaceuticals in low-flow dry seasons sustain selection prior to storm-driven dispersal (Mariano et al., 2023).

**FIGURE 1:**
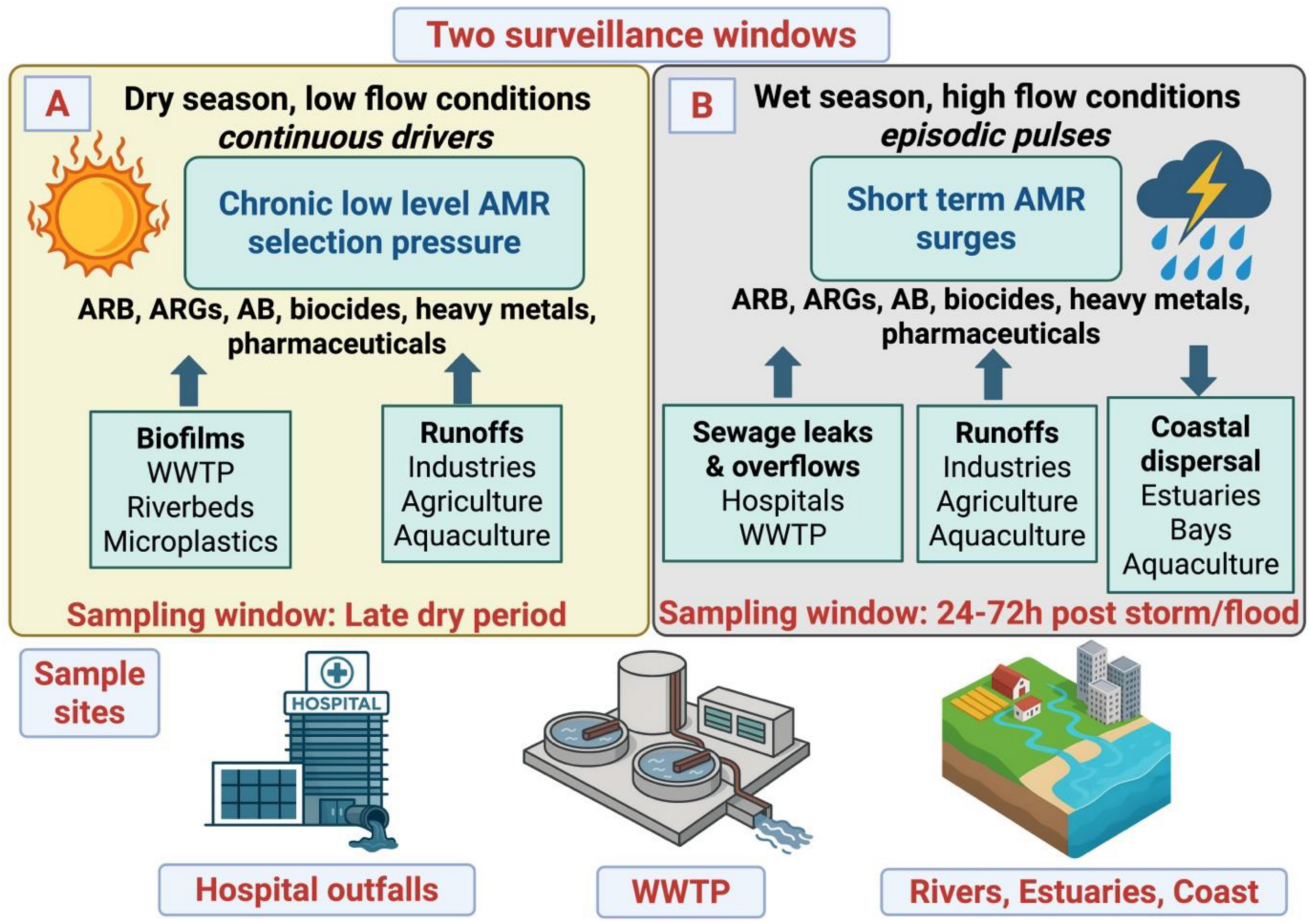
Two window climate AMR surveillance framework. Schematic contrasts dry season, low flow “chronic selection” (biofilms; sub ppb ABs, heavy metals, biocides; ARB/ARG accumulation) with wet season, high flow “flood pulses” (episodic surges from sewage leaks/overflows and runoffs) and maps sampling windows: **(A)** late dry period and **(B)** 24–72 h post storm/flood. Routine sampling targets hospital outfalls, WWTP influent/effluent, and river-estuary/coastal sites enable cross-sector comparisons and trigger source tracking. ARB, Antibiotic Resistant Bacteria; ARGs, Antibiotic Resistance Genes; AB, Antibiotics; WWTP, Wastewater Treatment Plants. Created by biorender.com.

For a LMIC archipelago with partial sewage and combined drainage, the environmental AMR problem is both continuous and pulsed. Effective hospital pre-treatment can block point releases, but network-level leakage and storm mobilization continue to drive ARB and ARG dispersal from facilities and settlements into Manila estuaries, the Laguna Lake basin, and coastal waters. To understand this dispersal pattern, two surveillance windows are essential: (i) late dry seasons which captures chronic selection signals (biofilms and ppb level residues of pharmaceuticals, heavy metals, biocides, and antibiotics which select for and stabilize resistance between storm pulses), and (ii) 24–72 h post storm/flood period which captures flood driven AMR surges (e.g., overflows of hospital sewage, WWTP, rivers) (Figure 1). These windows will generate baseline evidence for an integrated response of sewerage capture expansion, stormwater separation, and reliable hospitals pretreatment.

## 7 Environmental monitoring and closing data gaps

In the Philippines, environmental and food-chain pathways of AMR remain understudied despite their importance in One Health transmission. To date, ARB studies across One Health sectors have focused on *E. coli*; however, systematic monitoring of ESKAPE pathogens is still lacking. Moreover, existing studies vary in methodology—using different culture media (e.g., general, selective, or chromogenic media such as CHROMagar) leading to fragmented results and hindering cross-sector comparison. Proposed standardized protocols such as primary isolation on non-selective media (e.g., Tryptic Soy Agar, blood agar) should be used for unbiased rates, and optional secondary screening (e.g., CHROMagar ESBL) reported separately from primary rates. Taxonomic identification could be performed using MALDI-TOF or validated biochemical panels/assays (e.g., Vitek 2), and AST should be CLSI aligned (most commonly reported standards adopted in Philippine studies) to include 3GC, carbapenems, fluoroquinolones, and colistin.

To address culture limitations, advanced molecular approaches, such as high-throughput qPCR (HT-qPCR), targeted qPCR, WGS, and metagenomics can quantify ARGs, BMRGs, and mobile genetic elements (MGEs: integrons, plasmids, transposons) that drive and disseminate AMR (Ng et al., 2017; Tan et al., 2015). HT-qPCR, in particular, enables simultaneous detection and quantification of hundreds of ARG and MGE targets, yielding a rapid, high-resolution snapshot of resistance patterns across wastewater, livestock effluents, aquaculture, surface waters, and clinical samples, supporting cross-sector comparisons identifying hotspots and transmission pathways where culture-based monitoring is patchy (Knight et al., 2024; Silvester et al., 2025; Zhang et al., 2021). Table 3 provides recommendations for a structured two-tier molecular surveillance approach for implementation at regional hubs for routine screening, and national nodes for confirmatory source attribution. Tier 1 (HT-qPCR at regional hubs) proposes rapid, high-throughput screening of priority ARG/MGE panels to flat hotspots, and storm pulse spikes, which enables cross-sector comparability. Tier 2 (metagenomics/WGS at national nodes) provides depth in resistome/plasmid context and source tracing (triggered by Tier 1 thresholds).

**TABLE 3:** A two-tier molecular AMR surveillance approach for implementation at regional hubs and national nodes in the Philippines.

Framework component	Tier 1 (regional hubs)	Tier 2 (national nodes)
Platform	HT-qPCR	Shotgun metagenomics, target isolates for WGS
Purpose	Routine screening across sectors for baseline surveillance and cross-site comparison	Complete overview of resistome profiles across sites, including information on BMRG, MGE, host taxonomy, outbreak linkages, source attribution
Data Outputs	200–300 ARG/MRG priority targets (e.g., *bla*_NDM_, *bla*_CTX_, *bla*_KPC_, *mcr*, *vanA/B*, *intI*1, qacEΔ1) with absolute or relative abundances reported	ARG-MGE co-occurrence, plasmid typing, phylogenic diversity of sites (dependent on bioinformatic analyses)
Turnaround time	1–2 days	7–10 days (dependent on downstream computational analysis)
Cost*	∼$200–300/sample (200 gene targets)	∼$100–250/sample (10GB of data)
Benefits of implementation	AMR baseline establishment, detection of critical AMR markers across different sites (e.g., *bla*_NDM_, *bla*_CTX_, *bla*_KPC_, *mcr*), tracking AMR changes during dry-seasons and storm pulses	Triggered by Tier 1 flag (high detection of AMR marker), followed by targeted tracking along wastewater/animal sources river estuary route
AMR surveillance output	Routine reporting of ARG/MGE/BMRG indices and thresholds to warrant triggering Tier 2	Providing national source of pathways/maps for AMR intervention strategies.

*Dependent on volume of samples, costs in USD.

## 8 AMR risk assessment frameworks, a policymaking toolkit

Environmental AMR risk assessments are based on determinants such as AB, ARB, and ARGs, BMRG, vectors for HGT, and other environmental covariates that influence dissemination (e.g., weather, temperature, river flow). Philippine research on environmental AMR risk mainly uses two approaches: risk quotients (RQ) that compare measured antibiotic residues with aquatic PNEC, and culture based MAR indices for ARB. These approaches have been applied in surface waters (dela Peña et al., 2022; Palmares et al., 2024; Quadra et al., 2023; Van et al., 2021), wastewaters (dela Peña et al., 2022; Quadra et al., 2023), aquaculture (Rolando et al., 2022; Supnet et al., 2020) and groundwater settings (Recalcar et al., 2025). These methods are useful for prioritization and surveillance but do not quantify human health risk.

Table 4 summarizes the risk approaches/frameworks that are currently available and could be adopted in AMR studies in the Philippines. The MAR index remains one of the most widely used tools due to its cost-effectiveness and direct application, though it has limitations in capturing the complexity of resistance and cross-resistance (Goh et al., 2024; Krumperman, 1983). More recent approaches include integrative quantitative microbial risk assessment (QMRA)-disability adjusted life years (DALY), which estimates the human health burden of contact with ARB contaminated environmental waters (Goh et al., 2023; Heida et al., 2025; Schoen et al., 2021). Further to this, a modeling framework that integrates data from ARB and ARG has been developed to evaluate the relative burden of AMR, enabling comparative risk assessment across different locations, and thereby facilitating the identification of potential hotspots (Goh et al., 2022). Other tools such as ARG Risk Ranker (Zhang et al., 2021), MetaCompare 2.0 (Rumi et al., 2024), and Resistance Readiness Condition (RESCon) (Martinez et al., 2015) employ the use of resistomes identified in metagenomes to quantify human health and ecological resistome risks, whereas the Comparative AMR Risk Index (CAMRI) utilizes ARG prevalence data from qPCR or HT-qPCR, incorporating both abundance and risk coefficients (Goh et al., 2024). The RQ approach, frequently used in environmental chemical risk assessment has been adapted for antibiotics but faces challenges due to absence of threshold for certain emerging chemicals (Bengtsson-Palme and Larsson, 2016). The choice of AMR risk assessment framework depends on the intended outcome, whether it is used for identifying hotspots, estimating human health burden, or ranking high-risk ARGs for tracking across the One Health sectors. The Philippines National Action Plan (PNAP) on AMR 2024–2028 prioritizes research translation and explicitly supports risk-assessment studies on indirect exposure to AMR via animal consumption and environmental pathways (Strategic Objective 6, SO6), making quantitative risk evaluation central to evidence-informed policy and communication.

**TABLE 4:** Comparison of different AMR risk approaches/frameworks within the Philippine context.

Approach/framework	Data input	Data output	Key strengths	Examples of primary use cases	Level of implementation
HHRA for AMR^1^	ARB/ARG specific prevalence in water/food, exposure scenarios/frequencies, local dose-response	Infection risk estimates in humans	Regulatory, structured	Floodwater and drinking water exposure; recreational risk screening	At local government units (LGU), regional, national levels
PNEC thresholds^2^	Measured antibiotic concentrations, PNEC thresholds (ng/L–μg/L)	Identify sites that exceed safe limits	Simple, quantitative, antibiotic hotspot identification	Identify hotspots of antibiotic contamination in environmental waters (e.g., rivers, canals, estuaries) near hospitals, WWTPs, animal sources	Regional and national levels *Requirement:* Access to LC-MS/MS, standards and protocols
Integrated QMRA-DALY^3^	ARB prevalence, hazard and exposure data, dose and dose-response, additional burden due to resistance, local population and exposure, DALY parameters	DALY burden of AMR exposure	Outputs in DALYs, policy-relevant	Estimate burden of human contact with contaminated environmental waters, prioritize interventions (chlorination, clean ups)	Regional and national levels*m Requirement:* Analysis, framework support, national datasets
MAR index^4^	Isolates tested against antibiotic panels	MAR index, where MAR ≥ 0.2 = high-risk source (range of 0–2)	Low cost, simple indicator	Identify hotspots of ARB contamination in the environment (e.g., rivers, canals, estuaries) and track trends	At LGU, regional, national levels
RESCon^5^	Metagenomes/isolate data, ARG presence, mobility genes, pathogen hosts, metadata	Resistance Readiness Condition (RESCon) scores for ARGs that are likelihood to impact health, RESCon 1 = high risk, RESCon 7 = lowest risk	Conceptually simple, ARG-level prioritization	Rank high-risk ARGs (e.g., *bla*_NDM_, *bla*_KPC_, *mcr-1*, *bla*_CTX–M_, *vanA*) across different environments to support AMR surveillance panel design	Regional and national levels *Requirement:* Bioinformatic capacity
ARG Risk Ranker^6^	Metagenomes/genomes, metadata	Abundance of ARGs, ARG classification, contribution of each risk rank	Robust ARG prioritization	Select standardized Tier 1HT-qPCR targets for comparison across environmental waters	Regional and national levels *Requirement:* Bioinformatic and computational capacity
MetaCompare 2.0^7^	Metagenomes, metadata	Human-health and ecological resistome risk	Separates human and ecological risk	Track and create risk maps from environmental sources	Regional and national levels *Requirement:* Bioinformatic and computational capacity
CAMRI^8^	ARG prevalence, ARG risk rank (risk coefficients for the Philippines)	Risk indices enable comparative assessments across environments	Risk index that considers both likelihood (abundance of ARG) and severity (risk coefficient)	Compare AMR risks in aquaculture intensive waters	At LGU, regional, national levels

References:

^1^(Ashbolt et al., 2013);

^2^(Bengtsson-Palme and Larsson, 2016);

^3^(Goh et al., 2023);

^4^(Krumperman, 1983);

^5^(Martinez et al., 2015);

^6^(Zhang et al., 2021);

^7^(Rumi et al., 2024);

^8^(Goh et al., 2024).

## 9 Recommended priority actions aligned to the Philippines national action plan

In alignment with the PNAP-DRIVE (Driving Responsive and Innovative Participation of Vulnerable Sectors toward Empowerment in Local Governance) SO6, we outline the following priority actions for stakeholders across government, academe, private sector, and civil society to strengthen One-Health AMR governance (Table 5).

**TABLE 5:** Priority actions aligned to PNAP-DRIVE strategic objective 6.

Immediate	Mid-term
Task: Launch two surveillance windows (late-dry period; 24–72 h post storm/flooding event) at hospital outfalls, WWTP influent/effluent, rivers, estuaries, and log overflows Stakeholders: DENR-EMB, LGUs/water utilities, DOH (regional), hospital administrations	Task: Implement storm auto sampling paired with rainfall/river-stage logs, Stakeholders: DENR-EMB, LGUs/utility board, DOH-ARSP
Task: Issue shared standard operating procedure (SOPs) for non-selective primary culture; and report selective/chromogenic data separately Stakeholders: DOH-ARSP, DENR-EMB, BAI/BFAR	Task: Scale Tier 1 hubs to cover more regions and activate Tier 2 nodes (metagenomics/WGS) using Tier 1 ARG triggers Stakeholders: DOH-RITM, DOST/PCHRD, DENR-EMB, BAI/BFAR
Task: Pilot Tier 1 HT-qPCR at regional hubs Stakeholders: DOH-RITM, academe, DENR-EMB/LGUs	Task: Activate AMR molecular tracking pipelines for plasmid/clone dissemination (Tier 2 metagenomic/WGS), implement AMR risk assessment frameworks Stakeholders: DOH-RITM/Tier 2 nodes, academe, DENR-EMB
Task: Run AWaRe compliance audits in hospitals Stakeholders: Hospitals, DOH regional	Task: Include slaughterhouses and aquaculture sentinels to both surveillance window to harmonize reporting Stakeholders: BAI/BFAR, LGUs, DENR-EMB
Task: Require farm AMU log books (class, dose, duration, species) overseen by veterinarians Stakeholders: BAI, LGU veterinarians	Task: Link Tier 1 signals to targeted investigations and disseminate public updates Stakeholders: DOH-RITM/Tier 2, DENR-EMB, LGUs

DOH, Department of Health; ARSP, Antimicrobial Resistance Surveillance Program; RITM, Research Institute for Tropical Medicine; DENR, Department of Environment and Natural Resources; EMB, Environmental Management Bureau; DA, Department of Agriculture; BAI, Bureau of Animal Industry; BFAR, Bureau of Fisheries and Aquatic Resources; DOST-PCHRD, Department of Science and Technology-Philippine Council for Health Research and Development; AMU, Antimicrobial usage.

## 10 Conclusion

In the Philippines, *E. coli* isolated from poultry and swine show > 25% resistance to 3GC, > 73% to co-trimoxazole and > 44% to ciprofloxacin ( > 44%), signaling a clear farm to fork zoonotic risk (Gundran et al., 2020; Gundran et al., 2019). Critical data gaps persist outside clinics with non-clinical ESKAPE monitoring sparse. AMR is a cross-sector public health challenge that extends beyond hospitals into agriculture, and environmental reservoirs, with climate hazards amplifying spillovers. Clinically relevant lineages harboring plasmid-borne *bla*_NDM_, *bla*_KPC_ have been detected in hospital sewage and nearby rivers possibly linked to sewer leakages or overflows (Suzuki et al., 2020). The Philippines is an archipelago with a river-to-coast “pulse” hydrology driven by intense monsoon rains and typhoons. This triggers rapid flushes of antibiotics, ARB, and ARGs from on-site sanitation (septic tanks), combined sewers, and livestock/aquaculture drains into rivers and near-shore fisheries. The convergence of low sewerage coverage, dense floodplain settlements, and aquaculture at river mouths magnify storm/flood driven changes in AMR loads and exposure risks, making AMR a double pronged health-security and climate-resilience challenge. We recommend translating these risks into operational frameworks: (i) implementing a two-window, storm/flood-triggered surveillance (late-dry baselines and 24–72 h post-storm) at outfalls/WWTP/rivers/estuaries; (ii) two-tier molecular AMR surveillance system; (iii) implementation of AMR risk assessments as a policymaking toolkit (from data generated from two-tiered system; (iv) setting up sentinel hotspots and SOPs for monitoring (to close AMR data gaps); and (v) governance aligned to PNAP-DRIVE SO6. Climate change through droughts and increasingly frequent storm and flood pulses will intensify AMR vulnerabilities, disease transmission, and outbreak risks. We recommend mobilizing a two-window surveillance approach: late-dry and 24–72 h post storm as an immediate step to identify sources of sewer overflows and high-risk discharges, as well as standardizing SOPs for AMR methodologies for both culturing and molecular identification.
